# Apolipoprotein B and non-high-density lipoprotein cholesterol reveal a high atherogenicity in individuals with type 2 diabetes and controlled low-density lipoprotein-cholesterol

**DOI:** 10.1186/s12944-020-01292-w

**Published:** 2020-06-06

**Authors:** Liliana Fonseca, Sílvia Paredes, Helena Ramos, José Carlos Oliveira, Isabel Palma

**Affiliations:** 1 Endocrinology Department, Centro Hospitalar e Universitário do Porto, Largo Professor Abel Salazar, 4099-001 Porto, Portugal; 2 Endocrinology Department, Hospital de Braga, Sete Fontes, São Victor, 4710-243 Braga, Portugal; 3 Clinical Chemistry Department, Centro Hospitalar e Universitário do Porto, Largo Professor Abel Salazar, 4099-001 Porto, Portugal

**Keywords:** Dyslipidemia, ApoB, LDL-c, CVD, CV risk, Non-HDL cholesterol, T2DM

## Abstract

**Background:**

Lipid-lowering therapy is guided by Low-density-lipoprotein cholesterol (LDL-c) levels, although the cardiovascular disease (CVD) risk could be better reflected by other lipid parameters. This study aimed at comparing a comprehensive lipid profile between patients with type 2 diabetes mellitus (T2DM) with LDL-c concentration within and above target.

**Methods:**

A comprehensive lipid profile was characterized in 96 T2DM patients. The European Society of Cardiology/European Atherosclerosis Society (ESC/EAS) 2016 and 2019 Guidelines for the Management of Dyslipidemias were used to define LDL-c targets.

**Results:**

In this population, only 28.1 and 16.7% of patients had mean LDL-c levels within target, as defined by the 2016 and 2019 guidelines, respectively. Applying the 2016 guidelines criteria, in patients with LDL-c within target, 22, 25 and 44% presented non-high-density lipoprotein cholesterol (non-HDL-c), Apolipoprotein B (ApoB) and oxidized LDL-c levels above the recommended range, respectively, whereas according to the 2019 guidelines criteria, 50, 39 and 44% of the patients with LDL-c within target had elevated high-density lipoprotein cholesterol (HDL-c), ApoB and oxidized LDL-c levels, respectively. LDL-c was strongly correlated with non-HDL-c (*r* = 0.850), ApoB (*r* = 0.656) and oxidized LDL-c (*r* = 0.508). Similarly, there was a strong correlation between non-HDL-c with both ApoB (*r* = 0.808) and oxidized LDL-c (*r* = 0.588).

**Conclusions:**

These findings emphasize the limitations of only considering LDL-c concentration for cardiovascular (CV) risk assessment. Targeting only LDL-c could result in missed opportunities for CV risk reduction in T2DM patients. These data suggest that non-HDL-c, ApoB and oxidized LDL-c levels could be considered as an important part of these patients’ evaluation allowing for a more accurate estimation of CV risk and hopefully better management of these high-risk patients.

## Background

Diabetes mellitus is a risk factor for cardiovascular disease (CVD) and has been associated with 2- to 4-fold higher mortality [1–5]**.** In addition to glycemic control, the cornerstone of type 2 diabetes mellitus (T2DM) patients treatment is the management of the several cardiovascular risk factors that commonly are present in these patients. Dyslipidemia is common in T2DM patients and there is strong evidence that lowering cholesterol levels improves cardiovascular outcomes, even in patients with apparently adequate lipid profiles [6].

Low-density-lipoprotein cholesterol (LDL-c) is recommended as the primary target to guide lipid-lowering therapy [7]. However, there is evidence that other lipid parameters are also relevant in order to reduce coronary heart disease (CHD) events [8, 9], such as non-high-density lipoprotein cholesterol (non-HDL-c) and Apolipoprotein B (ApoB) which were considered secondary targets for lipid-lowering therapy according to the 2016 European Society of Cardiology/European Atherosclerosis Society (ESC/EAS Guidelines) [7]. It has been suggested that non-HDL-c or ApoB may provide a more accurate estimation of CHD risk than LDL-c [8, 9]. In 2019, the ESC/EAS Guidelines [5] recommended that, when evaluating patients with diabetes, metabolic syndrome, obesity, high triglyceride concentration or very low LDL-c levels, non-HDL and ApoB could be preferred in order to estimate CV risk. These guidelines also updated LDL-c targets and risk categories, classifying all T2DM patients with moderate, high or very-high CV risk.

T2DM patients present an atherogenic dyslipidemia characterized by increased triglycerides, decreased HDL particles, and LDL levels overlapping those of non-diabetics. Nevertheless, they also present small and dense LDL particles that are highly atherogenic and strongly increase these patients’ CV risk. Oxidized lipids in circulating LDL-c are also shown to be strongly associated with coronary atherosclerosis, arterial dysfunction, and mortality [10–12]. Therefore, when evaluating a diabetic patient with a basic lipid profile, which usually determines LDL-c, total cholesterol, triglycerides and HDL-c levels, its severe atherogenic susceptibility might not be reflected since in many patients these markers remain within the normal range [7], and thus, CV risk can be underestimated.

The aim of this study was to compare a comprehensive lipid profile between patients with type 2 diabetes mellitus (T2DM) with LDL-c concentration within and above target it was, according to the 2016 and 2019 guidelines.

## Patients and methods

A survey on our institution’s electronic medical records for patients with an extended lipid profile collected at a Portuguese University Hospital was performed. Patients followed in the Dyslipidemia outpatient department between January 2010 and September 2017 were selected. After analysis of the electronic medical records of 345 patients, 96 T2DM patients were included in this study. This study was approved by the local Ethics committee (150-DEFI/149-CES). Due to the retrospective design of this study, consent to participate was waived by the Ethics Committee.

Diabetes mellitus was defined as plasma glucose ≥126 mg/dL in at least two measurements, glycated hemoglobin (HbA1c) ≥6.5% or prescription of any antidiabetic medication [13].

Patients were classified as smokers if they consumed at least 1 cigarette per week. A former smoker was defined as having quit smoking at least 6 months before assessment. Alcohol consumption was determined when patients reported drinking at least 10 g or 20 g of pure ethanol daily, for women and men respectively. Patients with an history of stroke and/or transient ischaemic attack were considered to have cerebrovascular disease.

Conditions that could interfere with the lipid profile such as genetic dyslipidemia, hepatic or renal moderate to severe disease, cancer, viral infection (hepatitis B or C, human immunodeficiency virus), genetic metabolic disease, hyper or hypothyroidism were considered exclusion criteria. Pediatric patients and pregnant women were also excluded as well as patients treated with drugs that could alter the lipid profile as a side effect, such as glucocorticoids.

In order to stratify risk categories, patients were classified as having “very high risk” and “high risk” according to the 2016 ESC/EAS Guidelines for the Management of Dyslipidemias [7]. LDL-c targets defined by the 2016 ESC/EAS Guidelines [7] were as follows: LDL-c < 70 mg/dL for very high-risk patients and LDL-c < 100 mg/dL for high risk patients. The same guidelines were used to define the targets of the remaining lipoproteins [7]: ApoB: < 80 mg/dL in very high risk patients and < 100 mg/dL in high risk patients; non-HDL-c: < 100 mg/dL in very high risk patients and < 130 mg/dL in high risk patients; ApoA1: low if < 120 mg/dL in men and < 140 mg/dL in women; lipoprotein (a) (Lp(a)) < 50 mg/dL. For the remaining parameters, the following laboratory reference values were used: oxidized LDL-c: 26–117 mg/dL and ApoB/ApoA1 ratio: 0.45–1.25. In the 2019 guidelines update, risk categories were reviewed and new targets were defined according to patient’s CV risk: LDL-c < 55, < 70 and < 100 mg/dL for very-high-, high-, and moderate-risk patients, respectively; ApoB < 65, 80, and 100 mg/dL for very-high-, high-, and moderate-risk patients, respectively; and, non-HDL-c < 85, 100, and 130 mg/dL for very-high-, high-, and moderate-risk patients, respectively.

Biochemical laboratory tests were conducted after an 8-h night fast. Lipid parameters included total cholesterol, HDL-c, triglycerides, ApoB, ApoA1, oxidized LDL-c and Lp(a). LDL-c levels were calculated through Friedewald’s formula [14]: LDL-c (mg/dL) = total cholesterol (mg/dL) − HDL-c (mg/dL) – triglycerides (mg/dL)/5, unless triglycerides ≥400 mg/dL, in which case direct LDL-c measurement was performed. Non-HDL-c was calculated by subtracting HDL-c to total cholesterol. Other parameters such as creatinine, uric acid, glucose and homocysteine were also evaluated. HbA1c was assessed in most diabetic patients. Creatinine, glucose, uric acid, total cholesterol, HDL-c and triglycerides were measured using an enzymatic colorimetric method, with intra and inter-assay coefficients of variation of < 2.8% and < 3.9%, respectively. These parameters were measured using an automated autoanalyzer (Cobas 8000, Roche Diagnostics, Mannheim, Germany). ApoA1, ApoB and Lp(a) were evaluated through immunoturbidimetry, with intra and inter-assay coefficients of variation of < 1% and < 2.4% for ApoA1, < 1.2% and < 3.2% for ApoB and < 3.7% and < 3.8% for Lp(a), respectively. These parameters were evaluated using an automated autoanalyzer (Cobas Integra 400, Roche Diagnostics, Mannheim, Germany). Oxidized LDL-c was measured by Sandwich Enzyme-Linked ImmunoSorbent Assay (Mercodia, Uppsala, Sweden), with intra and inter-assay coefficients of variation of < 7.3% and < 8.3%, respectively. Small and dense low-density lipoprotein-cholesterol (sd-LDL) particles were measured using an enzymatic colorimetric method (Randox Laboratories, UK) with intra and inter-assay coefficients of variation of < 3%. HbA1c was determined using an ion-exchange chromatography method (Variant II turbo, BioRad Laboratories, CA, USA), with intra and inter-assay coefficients of variation of < 0.78% and < 0.66%, respectively. Homocysteine was measured by nephelometry (Dimension Vista 500, Siemens Healthcare, Germany), with intra and inter-assay coefficients of variation of < 3.3% and < 8.2%, respectively.

### Statistical analysis

Statistical analysis was performed using IBM SPSS® version 21.0 and a *p* value below 0.05 was considered statistically significant. For continuous variables, distribution normality was tested through histogram observation, kurtosis and skewness analysis. Results are presented as mean value ± standard-deviation and median values (25–75 percentiles). The chi-square test and the Fisher’s exact test were applied to analyze differences between groups regarding categorical variables. The Student t-test for independent variables and the Mann Whitney test were used to compare continuous variables with normal and non-normal distribution between groups, respectively. Correlations were evaluated using the Pearson and the Spearman correlation test for continuous symmetrical and asymmetrical variables, respectively.

## Results

This study evaluated 96 patients with T2DM (56 men and 40 women) with a mean age of 58.9 ± 9.0 years, median diabetes duration of 10 years (IQR: 4–7) and mean HbA1c of 8.1 ± 1.9%. The characterization of the sample is presented in Table 1. Mean LDL-c levels were 102.4 ± 38.6 mg/dL. The majority of patients (*n* = 48/72, 66.7%) were under lipid-lowering drugs and statins were the most common (*n* = 44/72, 45.8%), 90.4% (40/44) under moderate-intensity statins and the others under high-intensity statins. Ten (13.9%) patients were on treatment with fibrates and 4 (5.6%) with ezetimibe. Patients were not taking other drugs for treating dyslipidemia or any anti-diabetic drugs that could modify the lipid profile such as thiazolidinediones, glucagon-like peptide-1 agonists or sodium-glucose co-transporter-2 inhibitors. Most patients were under treatment with metformin (71.9%), followed by gliptins (53.1%), insulin (47.9%) and sulfonylureas (20.8%). According to the ESC/EAS guidelines, patients were divided in two groups: patients with LDL-c levels within target (*n* = 27) and LDL-c levels above target (*n* = 69) (Table 1).

**Table 1:** Comparison of clinical and laboratory variables between type 2 diabetes patients with LDL-c within and above target

	n	LDL-c within target (***n*** = 27)	n	LDL-c above target (***n*** = 69)	***p***
**Male** n (%)	27	16 (59.3%)	69	40 (58.0%)	0.908
**Age** (years)	27	59.8 ± 9.7	69	58.6 ± 8.8	0.571
Duration of diabetes* (years)	22	10.0 (3.5–15.3)	55	10.0 (5.0–19.0)	0.412
**Cardiovascular disease** n (%)	26	7 (26.9%)	68	15 (22.1%)	0.618
**Cerebrovascular disease** n (%)	26	5 (7.4%)	68	4 (15.4%)	0.256^1^
**Peripheral vascular disease** n (%)	27	5 (7.4%)	68	2 (7.7%)	0.955^1^
**Body mass index** (Kg/m^2^)	20	29.9 ± 3.7	56	30.6 ± 4.8	0.553
**Systolic BP** (mmHg)	24	142.0 ± 20.9	59	141.5 ± 18.3	0.928
**Diastolic BP** (mmHg)	11	71.8 ± 12.0	26	78.2 ± 15.4	0.229
**Waist circumference (M)** (cm)	12	102.1 ± 9.5	17	105.0 ± 11.9	0.492
**Waist circumference (F)** (cm)	3	96.0 ± 13.4	15	98.0 ± 12.7	0.438
Triglycerides* (mg/dL)	27	146.0 (106.0–295.0)	69	146.0 (108.0–207.5)	0.300
**HDL-c (M)** (mg/dL)	16	35.2 ± 9.68	40	41.7 ± 12.4	0.064
**HDL-c (F)** (mg/dL)	11	50.5 ± 10.7	29	46.6 ± 10.2	0.301
Glucose* (mg/dL)	22	137.0 (108.0–182.3)	59	151.5 (119.0–226.8)	0.062
**HbA1c** (%)	18	7.8 ± 2.6	48	8.2 ± 1.6	0.538
**Uric acid** (mg/dL)	21	5.1 ± 0.4	44	4.9 ± 0.2	0.656
**Homocysteine** (μmol/L)	20	11.4 ± 1.2	55	12.2 ± 0.8	0.632
**Statin use** n (%)	20	14 (70.0%)	52	30 (57.7%)	0.337
**Fibrates use** n (%)	20	2 (10%)	52	8 (15.4%)	0.716^1^
**Ezetimibe use** n (%)	20	3 (15%)	51	1 (2%)	0.065^1^
**Alcohol drinking** n (%)	8	1 (12.5%)	20	7 (35.0%)	0.371^1^
**Smoking** n (%)	21	2 (9.5%)	61	7 (11.5%)	0.805^1^
**Hypertension** n (%)	27	14 (51.9%)	68	56 (82.4%)	0.020

Data are presented as mean ± standard deviation, unless otherwise indicated by ∗ corresponding to data presented as median, 25th and 75th percentiles and ^1^ corresponding Fisher’s exact test. *LDL-c* Low-density lipoprotein cholesterol, *BP* Blood pressure, *HDL-c* High-density lipoprotein cholesterol, *M* male, *F* female

There were no statistically significant differences between the groups regarding gender, age, body mass index (BMI), waist circumference, HbA1c, triglycerides, HDL-c or glucose levels. No statistically significant differences between groups were found in relation to alcohol ingestion, smoking or use of lipid lowering therapy. Uric acid and homocysteine levels, predictors of new CV events, were not statistically different between groups. Patients with LDL-c levels above target had higher hypertension prevalence, although no significant difference was found when comparing the measurements of systolic and diastolic blood pressure.

The lipid profile of T2DM patients with LDL-c within and above target is presented in Table 2. Mean LDL-c concentration was 64.8 ± 14.7 mg/dL in patients with LDL-c within target and 117.1 ± 34.9 mg/dL in those with LDL-c above target (*p* < 0.001). The latter group also presented statistically significant higher total cholesterol, ApoB, non-HDL-c, ApoB/ApoA1 ratio and oxidized LDL-c levels. No statistically significant differences were found between the two groups regarding Lp(a) and ApoA1 particles. Despite having an LDL-c within the target defined by the 2016 guidelines, these patients presented elevated levels of other atherogenic lipoproteins. In fact, 22.2, 25 and 44.4% of patients presented non-HDL-c, ApoB and oxidized LDL-c levels, respectively, above the threshold for CV risk.

**Table 2:** Comparison of lipid profile between type 2 diabetes patients with LDL-c within and above target (2016 guidelines criteria)

	n	LDL-c within target	n	LDL-c above target	***p***
**LDL-c** (mg/dL)	27	65 (53–70)	69	109.0 (92.5–133.0)	< 0.001
**Total cholesterol** (mg/dL)	27	146 (125–162)	69	180.0 (74.3–107.8)	< 0.001
**ApoB** (mg/dL)	24	67.0 (58.8–79.0)	64	85.0 (74.3–107.8)	< 0.001
**ApoA1 (F)** (mg/dL)	8	167.5 (123.0–193.5)	27	155 (140–180)	0.556
**ApoA1 (M)** (mg/dL)	15	130 (102–143)	33	134.0 (116.5–139.5)	0.772
**ApoB/ApoA1 ratio**	22	0.54 (0.41–0.64)	60	0.63 (0.54–0.76)	0.004
Non-HDL-c* (mg/dL)	27	99.0 (85.0–118.0)	69	142.2 (119.5–166.5)	< 0.001
Oxidized LDL-c* (U/L)	27	115.7 (94.5–162.1)	69	150.8 (117.3–193.4)	0.007
Lipoprotein (a)* (mg/dL)	22	9.5 (4.0–34.5)	62	25 (5.75–58.10)	0.106

Data are presented as mean ± standard deviation, unless otherwise indicated by ∗ corresponding to data presented as median, 25th and 75th percentiles. *LDL-c* Low-density lipoprotein cholesterol, *ApoB* Apolipoprotein B, *ApoA1* Apolipoprotein A1, *M* male, *F* female, *Non-HDL-c* non-nigh-density lipoprotein cholesterol

After reclassification of the targets according to the 2019 guidelines, 16 (16.7%) patients were regarded has having LDL-c within target. Still, in these patients, 50, 38.8 and 43.8% presented elevated non-HDL-c, ApoB and oxidized-LDL-c, respectively. Of note, in patients with ApoB within target, 16.7% had elevated LDL-c. The lipid profile of the patients is exhibited in Table 3.

**Table 3:** **Comparison of lipid profile between type 2 diabetes patients with LDL-c within and above target** - **reclassification according to the 2019 guidelines**

	n	LDL-c within target	n	LDL-c above target	***p***
**LDL-c** (mg/dL)	16	55.5 (49–66)	80	104.5 (85.3–128.8)	< 0.001
**Total cholesterol** (mg/dL)	16	143 (125.8–150.8)	80	177 (159–213)	< 0.001
**ApoB** (mg/dL)	13	70 (54.5–86.5)	75	83 (70–106)	0.037
**ApoA1 (F)** (mg/dL)	3	160 (NA)	32	157.5 (140.3–180.8)	0.976
**ApoA1 (M)** (mg/dL)	9	135 (107.5–145.0)	39	134 (115–140)	0.792
**ApoB/ApoA1 ratio**	12	0.56 (0.39–0.68)	70	0.60 (0.51–0.74)	0.172
Non-HDL-c* (mg/dL)	16	95.5 (84.3–117.3)	80	138 (115.0–164.8)	< 0.001
Oxidized LDL-c* (U/L)	16	113.8 (76.2–160.2)	80	148 (113.9–185.5)	0.024
Lipoprotein (a)* (mg/dL)	10	6 (4.0–25.8)	74	24.5 (5.0–53.5)	0.232

Data are presented as mean ± standard deviation, unless otherwise indicated by ∗ corresponding to data presented as median, 25th and 75th percentiles. *LDL-c* Low-density lipoprotein cholesterol, *ApoB* Apolipoprotein B, *ApoA1* Apolipoprotein A1, *NA* not applicable, *M* male, *F* female, *Non-HDL-c* non-high-density lipoprotein cholesterol

LDL-c exhibited positive correlations with several lipid parameters. After Bonferroni correction, statistically significant positive correlations between LDL-c and total cholesterol (*r* = 0.895, *p* < 0.001), non-HDL-c (*r* = 0.850, *p* < 0.001), ApoB (*r* = 0.656, *p* < 0.001), ApoB/ApoA1 ratio (*r* = 0.291, *p* = 0.008) and oxidized-LDL-c (*r* = 0.508, *p* < 0.001) were found. Non-HDL-c was also significantly and positively correlated with ApoB (*r* = 0.808, *p* < 0.001) and oxidized-LDL-c (*r* = 0.588, *p* < 0.001).

## Discussion

Cardiovascular disease is the leading cause of death in patients with diabetes [15], making cardiovascular risk reduction a priority in these patients. LDL-c is recommended as a primary target to reduce CV risk by the 2016 and 2019 ESC/EAS Guidelines [7]. Nonetheless, even T2DM patients with LDL-c within the target range still have CV events, indicating there is residual CV risk that is unaccounted by LDL-c assessment. In accordance, the 2019 guidelines update have also highlighted the importance of ApoB and non-HDL-c determination in patients with diabetes. In fact, ApoB is also recommended to assess CV risk, particularly in patients with hypertriglyceridemia, diabetes, obesity, metabolic syndrome and very low LDL-c levels, and it can be used as an alternative to LDL-c for screening, diagnosis and management [5, 16]**.** The present study shows that in T2DM patients there are several atherogenic lipoproteins that remain elevated even in patients presenting an adequate LDL-c level. This study evidenced that 25 and 22.2% of T2DM patients had high ApoB and non-HDL-c levels, respectively, regardless of presenting an LDL-c concentration below the threshold for which treatment initiation is recommended. Moreover, when applying the LDL-c targets proposed by the 2019 guidelines, 38.8 and 50% of patients presented ApoB and non-HDL-c levels above target, respectively. Thus, as suggested by the 2019 guidelines, in patients with diabetes, LDL-c concentration seems to be insufficient to estimate the real CV risk. In this study, individuals with LDL-c levels above target had a higher rate of hypertension. Some studies have demonstrated that, when improving LDL-c and serum uric acid levels, pharmacologically or through lifestyle modification, the risk of developing hypertension is reduced [17, 18]. Cicero et al. demonstrated that in an overall healthy population sample, the contemporary presence of suboptimal serum uric acid and LDL-c values were associated with an increased risk of developing hypertension [17].

### ApoB and non-HDL

ApoB and non-HDL are not routinely determined, since they are considered secondary targets [7]. However, several studies have demonstrated an important role of both non-HDL and ApoB, when compared with LDL-c, regarding the occurrence of CHD events [19, 20]. In fact, ApoB levels have been shown to be associated with both arterial stiffness and cardiovascular disease [5, 21], whereas Cicero et al. demonstrated that ApoB levels were significantly associated with the carotid - femoral pulse wave velocity [22]. There is evidence that non-HDL cholesterol and ApoB may even be better predictors of CVD incidence among diabetic men than LDL-c [23]. This may occur because all major atherogenic particles originated in liver, namely, very-low density lipoproteins (VLDL) and intermediate-density lipoproteins have a apolipoprotein B100 molecule [24]. Therefore, ApoB measurement might be a direct proxy when assessing the number of atherogenic particles (LDL and non-LDL). Non-HDL-c has also been suggested as a better marker of CVD risk and coronary atherosclerosis [25]**.** Moreover, non-HDL-c seems to be associated with atherogenic dyslipidemia, insulin resistance, portal hyperinsulinemia and the metabolic syndrome phenotype [20, 24, 26–30]. Patients with higher triglyceride levels were shown to have lower LDL-c levels for any given ApoB concentration compared with subjects with lower triglyceride levels by Leroux et al. [31]. Thus, patients with hypertriglyceridemia, which is very frequent in T2DM patients, may present falsely lower LDL-c concentrations. However, these patients still have a high atherogenic risk, since ApoB levels remain elevated, even with decreased LDL-c levels [31]. Friedewald et al. recognized in their original work that, at lower LDL-c levels, even small errors in very low-density lipoprotein cholesterol estimation resulted in significant errors in LDL-c estimation [14] thus, in patients with LDL-c deemed appropriate by recent guidelines, the atherogenicity could be underestimated if only LDL-c is taken into account. To overcome this, the 2019 guidelines reinforce that, in patients with very low LDL-c levels, ApoB might be superior to LDL-c in assessing CV risk and guiding treatment. In this study, 22 and 25% of T2DM patients had high non-HDL-c and ApoB levels, respectively, even when presenting LDL-c within target. When analyzing data using the 2019 guidelines targets, the proportion of patients with elevated ApoB and non-HDL-c increased, despite having a LDL-c within target. These results suggest that in T2DM patients there is unaccounted CV risk if only LDL-c is considered, resulting in possible under treatment and missed CV benefits. This effect might be even more pronounced for lower LDL-c levels, in which the LDL-c/ApoB mismatch increases. Of note, in this study a number of patients (16.7%) with ApoB within target, according to the 2019 guidelines, still presented elevated LDL-c.

### HDL-c and ApoA1

Although an association between low HDL-c concentrations and an increased risk of T2DM has been shown at an epidemiological level [32, 33], serum HDL-c levels do not predict cardiometabolic risk, especially in T2DM [34]. HDL-c has a cardioprotective effect that has been attributed to its role in reverse cholesterol transport, its effects on endothelial cells, and antioxidant activity [35]. Similarly, HDL molecules also present anti-inflammatory, anti-apoptotic, vasodilatory, antithrombotic, and anti-infectious properties and can modulate the glucose metabolism directly [36]. Despite those observations, increasing HDL-c levels through medication have not shown to reduce CV events and HDL isn’t a primary target for CV risk reduction. In T2DM patients, HDL particles’ functions are impaired [37–40] and ApoA1 catabolism, a major HDL particle protein, is increased [37, 41]. In fact, Watts et al. [42] observed that ApoA1 production was increased in these patients in order to match the increased clearance. The mechanisms of increased ApoA1 clearance in T2DM are largely unknown, however the increased triglycerides fraction on HDL and the reduced adiponectin plasma levels observed in T2DM patients have been suggested to accelerate ApoA1 degradation [43]. It is also unclear whether hyperglycemia itself is linked to the increased clearance and may contribute to impaired HDL function, since lipoproteins are the primary targets of hyperglycemia-induced nonenzymatic glycation. In fact, glycated ApoA1 isolated from T2DM patients shows impaired self-association and lipid-binding abilities, as well as reduced stability [37]. In vitro studies demonstrated that human HDL combination with glucose resulted in reduced cholesterol efflux capacity [37, 38]. Sangeeta et al. [37] studied a T2DM patients cohort and demonstrated that despite normal HDL-c concentration, ApoA1 metabolism and function were clearly abnormal. T2DM patients had increased ApoA1 turnover and impaired cholesterol efflux and antioxidant activity of HDL. In summary, these findings suggest that serum HDL-c level is not a marker of reverse cholesterol transport, and neither HDL-c nor ApoA1 levels reflect HDL-c functionality [37, 38]. No differences were found in HDL-c levels between patients with LDL-c concentration within and above target. Low levels of ApoA1 also seem to independently associate with new onset of T2DM [44]. Nonetheless, in this study, patients evidenced normal ApoA1 concentrations and no differences were found between groups.

### ApoB/ApoA1 ratio

ApoB/ApoA1 ratio is a simple and accurate measurement to estimate CVD risk [45, 46]. Carnevale et al. (2011) described that a low ApoB/ApoA1 ratio reflects a less atherogenic lipid profile, regardless of LDL-c [46]. Several studies have also suggested that an elevated ApoB/ApoA1 ratio is a more powerful predictor than other lipid fractions for metabolic disorders, including T2DM [47–52]. Recently, it has been demonstrated that ApoB/ApoA1 ratio is independently associated with carotid atherosclerosis in T2DM patients with controlled LDL-c levels [53]. In this study, both groups presented a mean ApoB/ApoA1 ratio within the reference range. Park et al. [54] demonstrated that metabolic syndrome (MetS) patients showed an increased ApoA1 and suggested this was correlated with the presence of hypertriglyceridemia. Since T2DM patients frequently exhibited higher triglyceride levels, this could cause an increase in ApoA1 levels, decreasing the ApoB/ApoA1 ratio to normal range [54]. Patients with LDL-c levels above target, according to the 2016 guidelines, showed a significantly higher ratio than those within target. This observation didn’t hold when using the 2019 guidelines as reference, suggesting that, by lowering LDL-c concentration even more, there is a decrease in ApoB particles capable of reducing this ratio. Further studies with a higher number of patients are necessary to test this hypothesis.

### Oxidized LDL-c

In this study, 44.4% of T2DM patients and LDL-c within target had oxidized LDL-c above the reference range. Using the 2019 guidelines as reference, oxidized LDL-c concentration remained above the reference range in 43.8% of patients. Oxidized LDL-c has been associated with progression of atherosclerosis and CHD [55–57]. Holvoet et al. suggested that the predictive value of oxidized LDL-c seems to be additive to that of the Framingham global risk assessment score for CV risk [55]. Chronic hyperglycemia triggers the production of excess free radicals causing lipid molecules peroxidation in a chain reaction [58]. Malondialdephyde (MDA), oxidized LDL, oxidized LDL/LDL and oxidized LDL/HDL-c levels have been shown to be significantly elevated in T2DDM patients versus controls in several studies [59–61]. MDA is a recognized marker of lipid peroxidation and has been reported to modify ApoB, increasing the susceptibility of LDL-c to oxidation [56, 62]. Cicero et al. demonstrated a strong association of serum uric acid and oxidized LDL-c with carotid – femoral pulse wave velocity in individuals with normal and mildly impaired renal function. They suggested that serum uric acid and oxidized LDL-c should be monitored and managed as a marker of vessel stiffness [22]. In this study, even patients presenting LDL-c concentration within target showed elevated oxidized LDL-c levels, suggesting a high risk of atherosclerotic disease progression. This observation was maintained even after lowering the LDL-c targets according to the 2019 guidelines, indicating that oxidized LDL-c levels are not affected by LDL-c levels and could be a reliable index of atherogenicity in T2DM patients, regardless of the LDL-c status.

### Lipid parameters correlations

In T2DM dyslipidemia presents with a high concentration of free fatty acids and triglycerides, low HDL-c levels and increased sd-LDL particles and ApoB [63]. In this study LDL-c was positively correlated with total cholesterol, non-HDL-c and ApoB. Non-HDL also demonstrated a high correlation with ApoB and oxidized LDL-c, even stronger than for LDL-c. While ApoB and oxidized LDL-c assessment may not be routinely available, non-HDL-c measurement, as it is inexpensive and easy to obtain, could have a major role on identifying an atherogenic profile [29]. Additionally, non-HDL-c seems able to predict CVD over a wider range of triglyceride concentrations [29] surpassing Friedewald’s formula limitation, which is affected by triglycerides concentrations.

### Strength and study limitations

This study has some limitations that deserve comment. First, this is a small sample, especially regarding female gender. Secondly, it was a cross-sectional study with an associated bias not susceptible to rule out, especially the non-assessment of long-term outcomes, such as the occurrence of CVD according to lipid levels parameters. Nevertheless, these results can motivate a prospective study to evaluate if reducing other atherogenic lipoproteins, along with LDL-c, results in a greater reduction of CV events and mortality. This study also has strengths. A small number of studies have assessed non-conventional lipid parameters such as ApoA1, ApoB, Lp(a) or oxidized LDL-c in T2DM patients. Moreover, it is an important contribution to the characterization of a comprehensive lipid profile in patients with this condition. Finally, this study has important clinical implications since it confirms that, in T2DM, assessing solely LDL-c is insufficient for the correct estimation of the CV risk, resulting in a suboptimal treatment of these patients. There was a high mismatch LDL-c/ApoB thus, reinforcing the ESC/EAS 2019 guidelines recommendation of including ApoB measurement in patients with T2DM in order to better evaluate their atherogenic risk and guide adjustment of lipid-lowering therapy after achievement of the recommended LDL-c goal. Since a few patients with ApoB within target presented high LDL-c, LDL-c and ApoB should be additive regarding CV risk evaluation.

## Conclusions

LDL-c levels are currently accepted as the main therapeutic target for the prevention of CV events in T2DM patients. Nevertheless, even in patients with LDL-c considered within target, some patients still present atherosclerosis progression and CVD-related events. This study evidences that, although having LDL-c within target, patients presented elevated levels of molecules known for their atherogenic character, namely oxidized LDL-c, non- HDL-c and ApoB. The present study contributes to a better understanding of lipid metabolism in T2DM patients and has important clinical implications since it suggests that LDL-c seems to be insufficient to estimate CV risk. These data suggest that combining LDL-c with ApoB, oxidized LDL-c and non-HDL-c may add important information in order to better estimate CV risk in individuals with T2DM. Further studies should evaluate if intensive therapy, not only to reduce LDL-c but also other lipoproteins such as non-HDL-c, ApoB or oxidized LDL-c, has a beneficial impact on CVD and mortality.

## Data Availability

The datasets generated during and/or analysed during the current study are available from the corresponding author on reasonable request.

## Abbreviations

ApoA1: Apolipoprotein A1
ApoB: Apolipoprotein B
BP: Blood pressure
CHD: Coronary heart disease
CV: Cardiovascular
CVD: Cardiovascular disease
EAS: European Atherosclerosis Society
ESC: European Society of Cardiology
F: Female
HbA1c: Glycated hemoglobin
HDL-c: High-density lipoprotein cholesterol
LDL-c: Low-density lipoprotein cholesterol
Lp(a): Lipoprotein (a)
M: Male
MetS: Metabolic Syndrome
Non-HDL-c: Non-high-density lipoprotein cholesterol
SCORE: Systemic Coronary Risk Estimation
SD: Standard deviation
sd-LDL-c: Small and dense low-density lipoprotein-cholesterol
T2DM: Type 2 diabetes mellitus
VLDL: Very-low density lipoproteins
